# The American Public Health Association

**Published:** 1881-11

**Authors:** 


### THE AMERICAN PUBLIC HEALTH ASSOCIATION.

As previously announced in these columns, the ninth annual session of this organization will be held in Savannah on the 29th of November.

This association is the representative sanitary body of America, being composed of the leading sanitarians of the country, irrespective of vocation or professional aligment; including in its membership preachers, lawyers and business men, as well as physicians and scientists, it is intended to be popular in the widest sense of the term.

The objects of the Association are the promotion of public hygiene and the diffusion of sanitary knowledge among the people; and, it is claimed by those in a position to know, that it has already exerted more influence in this direction than any other body ever before organized. We are informed that a large attendance is anticipated, and it is especially hoped that this State will be fully represented at the meeting.

A cordial welcome, of course, awaits every guest of one of the most beautiful cities in the Union, famed alike for the refinement of its people and the elegance of its never-failing hospitality.

